# The importance of cardiac arrest registries

**DOI:** 10.1186/1757-7241-22-S1-A3

**Published:** 2014-07-07

**Authors:** Bryan McNally

**Affiliations:** 1 Emory University School of Medicine, Rollins School of Public Health, Atlanta, Georgia, USA

## 

Sudden cardiac arrest (SCA) is a global health concern. It is estimated that nearly half of all cardiovascular deaths worldwide are due to SCA resulting in an estimated 4 to 6 million cases each year [1,2]. Several countries have developed national out-of-hospital cardiac arrest (OHCA) registries for surveillance and quality improvement purposes including: Japan, Denmark, Singapore, Korea, Sweden, Ireland and many others are beginning to collect data. There are also collective efforts in Asia (PAROS), Europe (EuReCa), and the United States (CARES) underway [3-5]. Collecting data is an essential first step in determining the subsequent steps needed to strengthen the chain-of-survival within a community. It follows the business mantra that “it is hard to manage something if you don’t measure it”. Communities that don’t measure their OHCA outcomes are not only unable to gauge their performance but also lack a reference point to determine the impact of any implemented quality improvement efforts. In 2010, the American Heart Association recognized the importance of data collection for OHCA and identified the essential elements of a high quality resuscitation system that includes: measurement, benchmarking and providing feedback to influence change [6].

## Registries

### PAROS

The Pan-Asian Resuscitation Outcomes Study (PAROS) network was established in 2009 as an international, multicenter, prospective registry of OHCA events across the Asia-Pacific. The goal of the network “is to provide benchmarking against established registries and to generate best practice protocols for Asian emergency medical services (EMS) systems, to impact community awareness of pre-hospital emergency care, and ultimately to improve OHCA survival” [3]. To date the registry includes a population base of over 89 million with 9 countries being represented.

### EuReCa

In 2008, the European Resuscitation Council set up a working group with the goal of developing a common European Registry of Cardiac Arrest (EuReCa) and to be considered for use as a central tool for quality management in resuscitation. A pilot was performed and it was felt that it might best benefit those countries and regions that had not already set up registries of their own [4].

### CARES

In 2004, the US Center for Disease Control and Prevention developed the Cardiac Arrest Registry to Improve Survival (CARES) program https://mycares.net with the Department of Emergency Medicine at the Emory University School of Medicine. The registry evaluates OHCA of non-traumatic etiology for patients that receive resuscitative efforts, including CPR and/or defibrillation. Participating sites include data from three sources that allow for a patient centric outcome to be measured by linking data from: EMS providers, dispatch centers and hospitals. In the US the majority of persons who experience an OHCA do not receive bystander-assisted CPR or other time sensitive interventions that have been proven to increase survival rates (e.g. defibrillation).

CARES was developed as a low cost, high impact public health surveillance system to help identify opportunities for improvement in OHCA care. It was designed from the onset to make the process of data collection as simple and easy as possible. Because CARES data are collected in a uniform manner, the system enables benchmarking and continuous quality improvement in communities of any size. Nearly half of OHCA events are witnessed, so efforts to improve survival should consider the timely and effective delivery of interventions by bystanders and emergency providers [5].

## Global data trends and effectiveness

A recent data published from the Danish registry suggests an improvement in survival over time with a corresponding increase in both bystander CPR and defibrillation use [7]. The All Japan Utstein Registry has also previously shown an increased survival trend over time and an analysis currently under review for CARES data suggests the same as well [8]. These improved survival trends are most likely multifactorial in nature and since they are based on observational data it is impossible to pinpoint with certainty what is responsible for these trends. An improved survival trend over time is important to acknowledge but equally important is understanding the limitations of registry data.

**Efficacy** is the extent to which a treatment has the ability to bring about its intended effect under **ideal circumstances**, such as in a randomized clinical trial. **Effectiveness** is the extent to which a treatment achieves its intended effect in the **usual clinical setting**. Efficacy *is not the same* as effectiveness. A treatment is effective if it works in real life in non-ideal circumstances. Effectiveness cannot be measured in controlled trials, because the act of inclusion into a study is a distortion of usual practice. Just as a randomized control trial can’t be used to answer an effectiveness question an observational study can’t be used to answer an efficacy question. It is important to understand that both study designs are needed to help advance our understanding of OHCA as neither alone can answer both treatment and performance questions. This dual requirement is highlighted below.

“It is an irony that drugs are licensed for use almost exclusively on the results of controlled trials, yet they are withdrawn from use because of observational data that would not be acceptable to licensing authorities” [9].

In an editorial on the limitations of clinical trials in cardiac arrest, author Arthur Sanders acknowledged that “there are fundamental tensions between the principles of randomized trial design and the practice of resuscitation that make the conduct of any clinical trial of out-of hospital cardiac arrest challenging”. He added that “Randomized, controlled trials may not be the best strategy for making progress in the management of a public health problem” and considered that an alternative strategy would be to use a continuous-quality-improvement model.” He further concluded that “there are inherent limitations in even a well-designed, carefully executed clinical trial in advancing resuscitation science. It is therefore important that we reassess the role of clinical trials and alternative strategies in improving the rate of survival from cardiac arrest. The goal of resuscitation is saving lives; research helps achieve this goal but is not the goal itself” [10].

## The future

OHCA registries will continue to play an important role in the future at both the community and country level. Benefits will include: determining clinical **outcomes; uniform benchmarking**; identifying opportunities for improvement and tracking the diffusion of new therapies; and promoting accountability and answering effectiveness research questions.

Additional supplemental data elements could also be considered to enhance registries to: track AED locations in communities in real time to help a dispatcher link a caller with a nearby device; include CPR quality metrics in an effort to improve clinical performance by focusing on compliance with recommended guidelines; including telephone CPR data and quality improvement tools to ensure dispatchers recognize OHCA over the phone while minimizing the time from call receipt to instructions being provided and eventual first compression being performed. More robust hospital data could also be considered where resources allow to improve timely reperfusion and hypothermia treatment for OHCA patients when indicated.

OHCA data should be used as a starting point for improving a community based system of care by participating in a registry and quality improvement program but saving a life should continue to be the goal not proving how you did it.
